# Functional fingerprinting of human mesenchymal stem cells using high-throughput RNAi screening

**DOI:** 10.1186/s13073-015-0170-2

**Published:** 2015-05-17

**Authors:** Gerrit Erdmann, Michael Suchanek, Patrick Horn, Fabian Graf, Christian Volz, Thomas Horn, Xian Zhang, Wolfgang Wagner, Anthony D. Ho, Michael Boutros

**Affiliations:** German Cancer Research Center (DKFZ), Division Signaling and Functional Genomics and Heidelberg University, Department of Cell and Molecular Biology, Medical Faculty Mannheim, Im Neuenheimer Feld 580, D-69120 Heidelberg, Germany; Department of Medicine V, Heidelberg University, Im Neuenheimer Feld 410, D-69120 Heidelberg, Germany; RWTH Aachen Medical School, Helmholtz Institute for Biomedical Engineering, D-52074 Aachen, Germany

## Abstract

Mesenchymal stem cells (MSCs) are promising candidates for cellular therapies ranging from tissue repair in regenerative medicine to immunomodulation in graft versus host disease after allogeneic transplantation or in autoimmune diseases. Nonetheless, progress has been hampered by their enormous phenotypic as well as functional heterogeneity and the lack of uniform standards and guidelines for quality control. In this study, we describe a method to perform cellular phenotyping by high-throughput RNA interference in primary human bone marrow MSCs. We have shown that despite heterogeneity of MSC populations, robust functional assays can be established that are suitable for high-throughput and high-content screening. We profiled primary human MSCs against human fibroblasts. Network analysis showed a kinome fingerprint that differs from human primary fibroblasts as well as fibroblast cell lines. In conclusion, this study shows that high-throughput screening in primary human MSCs can be reliably used for kinome fingerprinting.

**Electronic supplementary material:**

The online version of this article (doi:10.1186/s13073-015-0170-2) contains supplementary material, which is available to authorized users.

## Background

Mesenchymal stem or stromal cells (MSCs) are multipotent adult stem cells capable of differentiating into cells of mesodermal origin such as bone, cartilage, muscle, connective tissue, and fat. They might play a role as a major cellular component of the bone marrow niche for hematopoietic stem cells [1]. MSCs were initially identified in the bone marrow but have been isolated from multiple tissues, including fat and amniotic tissue [2]. Due to their diverse differentiation potentials, the relative ease of their isolation from multiple tissues, the fact that they can be expanded and multiplied *in vitro*, and their immunomodulatory properties, MSCs are regarded as a promising tool for clinical applications. Indeed, MSCs are currently used in over 300 pre-clinical as well as clinical studies [3] and are being tested for applications in contexts such as acute myocardial infarction, liver failure, osteoarthritis, diabetes and accelerated wound healing [4–8]. Furthermore, MSCs possess substantial immunomodulatory activity — e.g., through expression of indoleamine 2,3-dioxygenase and other effector molecules — which can be used for treatment of graft-versus-host disease [9].

Despite initial promising results, progress has been hampered by the fact that standardization and characterization of MSCs remain insufficient. MSCs are notoriously heterogeneous, comprising cell subpopulations that might have unexpected effects or adverse side effects on the recipient [10]. The phenotypic and functional heterogeneity of MSC preparations from different laboratories has rendered a proper comparison of trials from different institutions impossible. Above all, no exclusive surface marker constellation for MSCs has been identified and the repertoire currently used to characterize MSCs has failed to irrevocably identify a marker for them.

Thus far, MSCs have been defined by their plastic adherent growth, their differentiation potential under specific conditions *in vitro*, and a panel of surface markers which should either be present (CD73, CD90, CD105) or absent (CD34, CD45, CD14 or CD11b, CD79a or CD19, HLA-DR), as proposed by the International Society of Cell Therapy [11]. However, these guidelines fail to account for MSC heterogeneity and are unable to discriminate between MSCs and fibroblasts [12, 13]. Furthermore, to fully exploit the therapeutic potential of MSCs in clinical trials and correlate molecular characteristics to clinical outcome, methods for more stringent characterization are crucial. Therefore, there is an urgent need to establish robust high-throughput approaches to define primary human MSCs from different donors and tissues as well as distinguish them from closely related cell types, such as fibroblasts.

Functional fingerprinting based on cell viability might complement current MSC characterization methods, such as surface marker expression and differentiation assays, to increase the repertoire for a better definition of MSCs. Cell viability can easily be quantified in a high-throughput manner and shows a good perturbation and dynamic range between different conditions and cell types [14]. High-throughput genetic screening approaches are broadly applied in model organisms and cells to delineate cellular pathways and identify components of cellular networks. RNA interference (RNAi) allows the systematic depletion of RNA transcripts and to analyze the influence of genes on diverse cellular processes. While such methods have been successfully applied in model organisms and in transformed cell lines, their use to dissect processes in primary cells has thus far been limited due to technical challenges. However, recent insights into genotype-to-phenotype relationships of complex traits in primary cells, such as MSCs, will allow an in-depth understanding of the functional properties of these cells. By adapting high-throughput methods originally developed for transformed cell lines, we have come up with strategies to quantitatively characterize and define the phenotype of human cell preparations [15–17]. Transferring established screening protocols from immortalized cell lines to primary cells is not trivial and technical bottlenecks include required cell numbers, susceptibility to small interfering RNA (siRNA) transfection, assay robustness and the issue of heterogeneity accompanying primary cells [17].

Primary MSCs can be expanded to large numbers *in vitro*, which is a prerequisite for high-throughput RNAi screening techniques. In this project, we have successfully established a high-throughput RNAi approach using primary MSCs from human bone marrow. Our protocols are robust against donor variability and hence this method is feasible for functional fingerprinting. Based on profiling of human kinases, we have shown that MSCs from bone marrow have distinct functional profiles compared with primary fibroblast and fibroblast cell lines. Finally, this technique provides a powerful tool to study and to broaden our understanding of primary adult stem cells and to analyze genotype-to-phenotype relationships.

## Methods

### Isolation and culture of MSCs from human bone marrow

Human bone marrow samples from the iliac crest were collected by bone marrow aspiration after written consent using the guidelines approved by the Ethics Committee of the Heidelberg University (348/2004). The mononucleated cell fraction was isolated after density gradient centrifugation using lymphocyte separation medium LSM 1077 (PAA Laboratories). After washing, mononucleated cells were re-suspended in low FCS culture medium consisting of low glucose Dulbecco's modified Eagle's medium (DMEM; PAA Laboratories) supplemented with 40 % (v/v) MCDB201 (Sigma), 2 % (v/v) fetal calf serum (FCS; HyClone), 2 mM L-glutamine (Sigma), 100 U/mL penicillin/streptomycin (Pen/Strep; Lonza), 1 % (v/v) insulin transferrin selenium (Sigma), 1 % (v/v) linoleic acid-albumin from bovine serum albumin (Sigma), 10 nM dexamethasone (Sigma), 0.1 mM l-ascorbic acid 2-phosphate (Sigma), 10 ng/mL of each PDGF-BB and EGF (PreproTech) and seeded in T75 vented filter cap tissue culture flasks (BD Biosciences) at a concentration of about 1 × 10^6^ cells/cm^2^. Medium was changed after 2–3 days to remove non-adherent cells. Initial colonies were separated and further cultured. After reaching 80 % confluence the cells were detached with 0.25 % Trypsin EDTA (PAA Laboratories), washed and seeded at a density of 10,000 cell/cm^2^ for expansion. MSCs were further characterized for their ability to differentiate towards osteogenic and adipogenic lineages and their immunophenotype with the Stemflow hMSC Analysis Kit (BD Biosciences). MSCs were used for experiments at passage 4.

### Culture of primary fibroblasts and fibroblast cell lines

Right atrial auricle specimen (0.5–1.0 g) was obtained as approved by the Ethics Committee of the University of Heidelberg (S-452/2009) and stored at 4 °C in high glucose DMEM (Gibco). Specimens were rinsed twice with ice-cold phosphate buffered saline (PBS) and cut into small pieces (1 × 1 × 2 mm). Fragments were digested with 0.05 % trypsin at 37 °C for 5 minutes. Specimens were then allowed to attach to the surface for 25 minutes before DMEM high glucose supplemented with 20 % (v/v) FCS and 100 U/mL Pen/Strep were added. Medium was change every other day and dishes were monitored daily for cellular outgrowth. Before outgrowth became confluent, primary fibroblasts were harvested and cultured at 37 °C and 5 % CO_2_ (standard conditions) in DMEM (10 % [v/v] FCS, 100 U/mL Pen/Strep). HFF1 (ATCC: SCRC-1041) and HS69 (ATCC: CRL-1635) fibroblast cell lines were cultivated in DMEM high glucose supplemented with 10 % (v/v) FCS and 100 U/mL Pen/Strep under standard conditions. Media were changed every other day and cells were passaged at 80–90 % confluency. At passage 3 fibroblasts were used for high-throughput RNAi screening.

### Characterization of MSC differentiation potential

To test their adipogenic differentiation potential, MSCs were cultured in medium consisting of DMEM high glucose media with 10 % (v/v) FCS, 2 mM L-glutamine, 100 U/ml Pen/Strep, 1 mM dexamethason (Sigma), 0.5 mM 1-methyl-3-isobutylxanthin (Sigma) and 10 mg/ml Insulin (Sigma). Medium was changed twice a week. Osteogenic differentiation was induced using DMEM low glucose with 10 % (v/v) FCS, 2 mM L-glutamine, 100 U/ml Pen/Strep, 100 nM dexamethason (Sigma), 200 mM L-ascorbic acid 2-phosphate (Sigma) and 10 mM B-glycerophosphate (Sigma). After 3 weeks the cells were stained with Oil red O (Sigma) for adipogenic and Alizarin red S (Sigma) for osteogenic differentiation. To check for the MSC-specific immunophenotype, surface marker expression was tested using the Stemflow hMSC Analysis Kit (BD Biosciences).

### High-throughput RNAi screening and cell viability assay

For cell-based screening, a human library for protein kinases (778 kinases) was arrayed, from the genome-wide siGENOME RNAi library (Dharmacon SMART pool 0.5 nmol; Thermo Fischer Scientific), in white 384-well LIA-Plates (Greiner) using a Biomek FX200 liquid handling system (Beckman Coulter). Each well contained 5 μl of a 250 nM pool of four siRNAs re-suspended in siRNA solution buffer (Dharmacon). Library siRNAs were spotted in columns 5–24; the remaining columns were used for controls. Rluc (P-002070-01) siRNAs where positioned at G04–J04, and positions F03–I03 contained siRNA pools targeting UBC (MU-019408-01). DharmaFECT1 (0.05 μl; 0.1 %) was mixed with 4.95 μl of RRMI and incubated for 10 minutes prior to addition of 10 μl of RPMI 1640 medium (Gibco). The transfection mix was distributed into the siRNA library plates. After 30 minutes, 200 cells/well (10,000 cells/cm^2^) were seeded into each well. Viability was quantified 72 h post-siRNA transfection by measuring total ATP amount with the luminescent-based CellTiterGlo (CTG; Promega) assay in a 384-well format. Cells were lysed through addition of 20 μl CTG reagent diluted 1:4 in fresh RPMI. The plates were incubated in the dark for 10 minutes and luminescence was measured for 0.1 s with a Mithras LB 940 plate reader (Berthold Technologies). Relative viability was determined by normalizing the measured relative light units (RLU) to the internal control of both untreated cells (NT) and cells transfected with scrambled or pGL3 siRNAs (Ctrl). Each donor was screened in duplicate and analyzed with cellHTS [18]. Each gene was scored on a multiplicative scale. The median of the log transformed values was calculated and variance was adjusted by experiment. Summarized replicates were determined and z-scores based on plate mean were calculated. Screening data were uploaded to the GenomeRNAi database and are publicly accessible under the accession number GR00342 [18].

### Cytometry and fluorescent microscopy

Seventy-two hours after siRNA transfection, cells were washed three times with PBS, fixed with 4 % (v/v) paraformaldehyde containing 0.02 % (v/v) Triton X-100 (AppliChem) at room temperature for 30 minutes and subsequently stained for 1 h with 1.25 μg/ml of Hoechst, to visualize DNA. Peak intensity, total peak intensity and number of Hoechst stained objects were measured on an Acumen Explorer microplate cytometer ex3 HCS (TTP LabTech), using the following parameters: Hoechst voltage, 550; sliding window, x = 1 μm y = 1 μm. In addition, a population manager was applied setting a minimal and maximum object size to exclude false positive objects. For MSC morphology studies, MSCs were blocked with 1 % bovine serum albumin and additionally stained for 1 h with Alexa Fluor 547 Phalloidin (1:3,000; Life Technologies) and fluorescein isothiocyanate (FITC)-conjugated α-tubulin (1:1000; Sigma). Images were taken using the BD Biosciences Pathway 855 imaging system. Three images were taken from each well at 10× magnification to cover approximately 60 % of the total well area. Images were analyzed using ImageJ (v.1.440). For each experiment at least one positive (UBC) and one negative control (pGL3) were used.

### Cell cycle analysis

Cells were reverse transfected with siRNAs 96 h before analysis at a confluence of 40 %. MSCs were collected by trypsination and stained with 200 μg/ml of propidium iodide (VWR), 0.1 % (w/v) sodium azide (Sigma Aldrich), 0.1 % (v/v) Triton-X100 (Sigma Aldrich) and 10 μg/ml RNAses (Qiagen) for 2–4 h at 4 °C. Single cells were analyzed for fragmented DNA, sub G1, S and G2/M peaks by fluorescence flow cytometry array (BD Biosciences). Analysis was performed with the FlowJo software 887 (Tree Star Software).

### Quantitative PCR

RNA was extracted according to the RNeasy Mini Kit protocol (animal cells vacuum/spin). The cDNA was prepared from 0.5–1.5 μg of total RNA, using the RevertAid H Minus First Strand cDNA Synthesis Kit (Thermo Fischer Scientific) and oligodT primer. cDNA (25 ng) was used for each quantitative PCR reaction on the Lightcycler480 (Roche), running the universal probe library system (UPL, Roche) in a 384-well format. *GAPDH* and *UBC* were used as reference genes for relative quantification.

### Statistical analysis

All data are represented as mean ± standard deviations. Statistical analysis was performed by unpaired two-tailed student's *T*-test. Results are considered significant for *p* ≤ 0.05 or *p* ≤ 0.01. In all experiments MSCs from at least three different donors were tested (N ≥ 3). Correlations were calculated using R/Bioconductor. Heatmaps were generated using the Multi Experiment Viewer (MeV v.4.8). Viability screening data were normalized to average of control siRNAs per plate and log_2_ transformed prior to uploading into MeV. Hierarchical clustering was performed with standard settings (optimizing leave structure). Differentiating gene groups were identified by *T*-test within MSCs and fibroblasts. On the identified clusters we performed post hoc power calculations using two-sample *T*-tests (G*Power 3.1.7) [19], assuming a significance level of 0.05 and a sample size of N = 2 for MSCs and N = 4 for fibroblasts. MSC1A and MSC1B were grouped because they are not biologically independent cell populations.

## Results

### High-throughput RNAi screening in MSCs

**Fig. 1:**
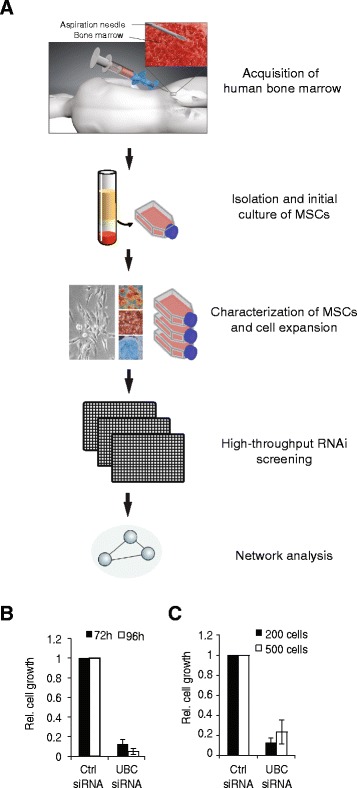
Workflow for high-throughput RNAi screening experiments in primary human MSCs. **a** Bone marrow is aspirated from healthy human donors and following density gradient centrifugation the mononuclear cell fraction is seeded into culture flasks. Initial MSC colonies are separated by their plastic adherence and expanded. MSCs are further characterized by immunophenotyping using fluorescence flow cytometry and their ability to differentiate towards adipogenic and osteogenic lineages. For high-throughput screening, MSCs were used at passage 4 and reverse transfected in cell culture plates. **b** Optimization of siRNA transfection conditions for assay time (at 200 cells/well) (**b**) and varying cell numbers (at 72 h) (**c**). Cell viability was determined using a CellTiterGlo assay. *Ctrl*, pGL3; *UBC*, siRNA against Ubiquitin C

To develop a highly standardized workflow for isolation and functional screening of MSCs from human bone marrow of healthy donors, we have optimized the transfection conditions with regards to transfection reagent, cell number and incubation time. After isolation, differentiation potential of the MSCs towards adipogenic and osteogenic lineages and expression of the surface markers CD73, CD90, and CD105 and non-expression of CD11b, CD19, CD34, CD45, and HLA-DR were confirmed. Cell preparations that fulfilled these criteria were used at passage 4 for high-throughput experiments (Fig. 1a). As a positive control we used siRNA against Ubiquitin C (*UBC*), a gene which is required for cell viability; as negative controls (pGL3 and scrambled siRNA) were used to test for toxic side effects of the reverse transfection procedure. These experiments established conditions whereby primary MSCs could be efficiently transfected by reverse transfection suitable for high-throughput RNAi experiments (Fig. 1b, c). In addition, we optimized conditions for secondary assays using laser scanning cytometry and fluorescent microscopy as readouts for cellular phenotypes (data not shown). Taken together, we have established a quantitative and efficient protocol for high-throughput functional analysis in primary MSCs.

**Fig. 2:**
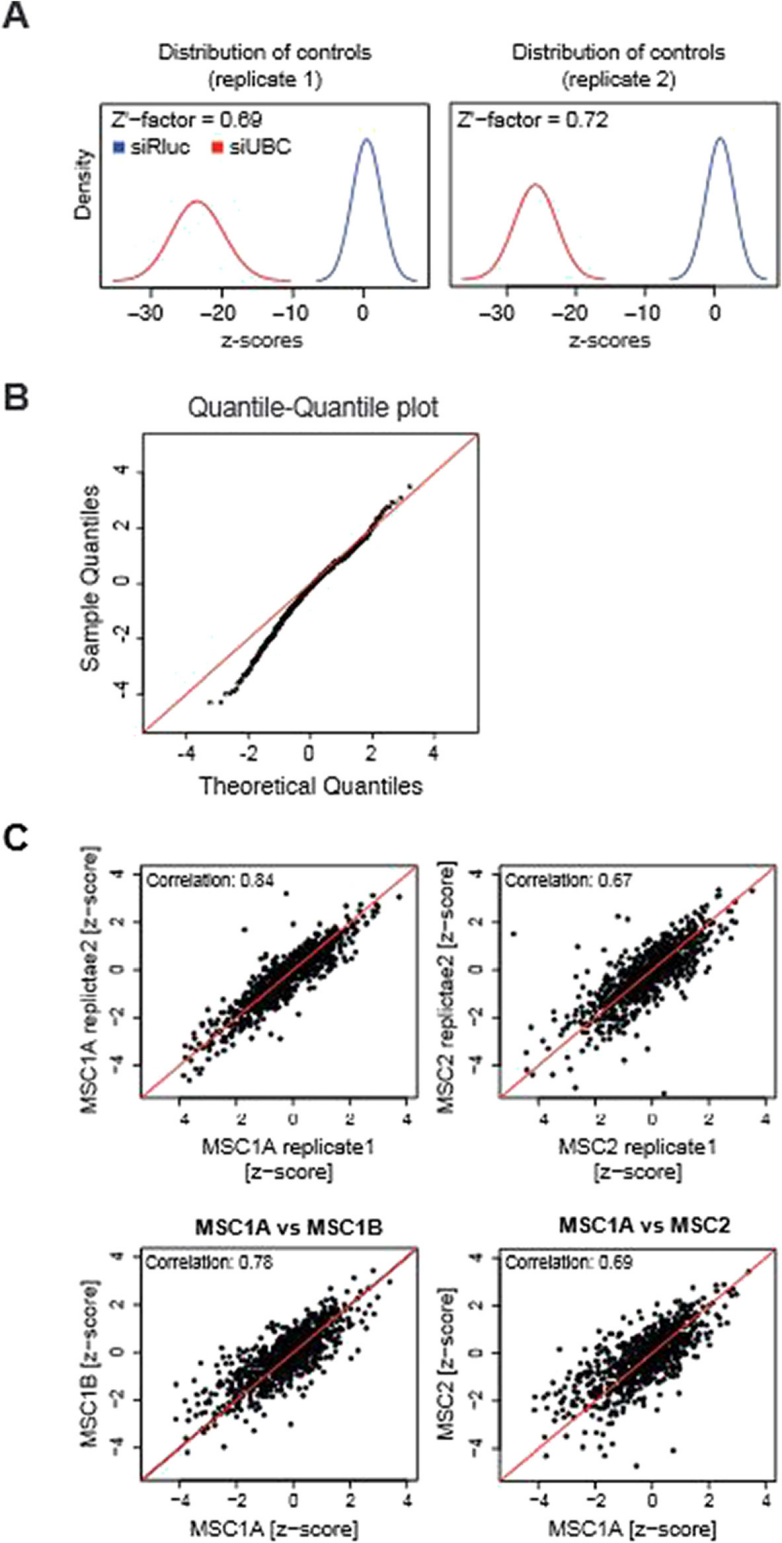
High-throughput screening in MSCs produces robust results between different primary cell preparations. **a** Relative distribution of positive *UBC* (red) and negative Rluc (blue) controls used in the kinome-wide screen based on their deviation from the screen mean (z-scores). Technical replicates from the same MSC donor are shown, both displaying the high dynamic range of viability effects detectable by the screen. **b** Probability plot of the screening results, comparing theoretical quantiles assuming normal distribution (horizontal axis) against actual results of one representative high-throughput screen (vertical axis). Values are plotted according to their calculated z-score. In the low end of the distribution screening results diverge from the linear pattern, indicating biologically significant changes in cell viability. **c** Correlation plots of z-scores between technical replicates of the same MSC preparation (MSC1A and MSC1A), two MSC preparations from the same donor (MSC1A and MSC1B), and MSCs from two different donors (MSC1 and MSC2) show high correlation between MSC preparations (Pearson correlation is indicated)

Next, we performed multiple RNAi screens against 778 kinases in the human genome (subsequently referred to as 'kinome'). In subsequent experiments, to exclude potential off-target effects, four independent siRNA designs targeting the same kinase of selected candidates were tested. Modification of the phosphorylation state of molecules strongly affects their characteristics, making kinases and phosphatases of utmost relevance for innumerable cellular pathways. To assess the reproducibility and heterogeneity of MSCs in high-throughput screening, we used two preparations of MSCs from different donors as well as two preparations of MSCs from the same donor, which were prepared and expanded independently. For each MSC preparation, siRNAs were screened in replicates and cell proliferation and viability were assessed 72 h after transfection. Each 384-well plate contained multiple negative (pGL3) and positive (siUBC) controls to assess transfection efficiency and variability. Data were analyzed using cellHTS2 [20] (see Methods; and Genome RNAi accession number GR00342). Analysis of controls showed that the high-throughput assay worked appropriately under screening conditions, with Z’-factors exceeding 0.5 (0.69 and 0.72, respectively; Fig. 2a), indicating that the assay in primary MSCs performs on par with high-throughput screenings in established cell lines [21, 22]. Overall, a quantile-quantile analysis [19] showed that a larger number of siRNAs induced impaired cell growth and viability phenotypes than expected from a normal distribution (Fig. 2b, below the red line), whereas fewer perturbations showed an increase in cell growth (Fig. 2b, above the red line).

We then assessed the comparability between independent replicate measurements and screening experiments performed in MSCs from different donors. We found that replicated screens in MSCs from the same donor showed high correlation (Pearson coefficient of 0.84; Fig. 2c, upper left panel), similar to experiments performed in HeLa or HCT116 cells (data not shown). The correlation between independent screens of MSCs from independent donors decreased to 0.72 and 0.69, respectively, which is still high for functional experiments.

In summary, these experiments provide evidence for the reproducibility of the isolation and high-throughput screening procedure and demonstrate that the heterogeneity reported for MSC isolation does not interfere with high-throughput screening even when cells from different donors were utilized.

### The kinome screens identified multiple proteins required for MSC growth

**Fig. 3:**
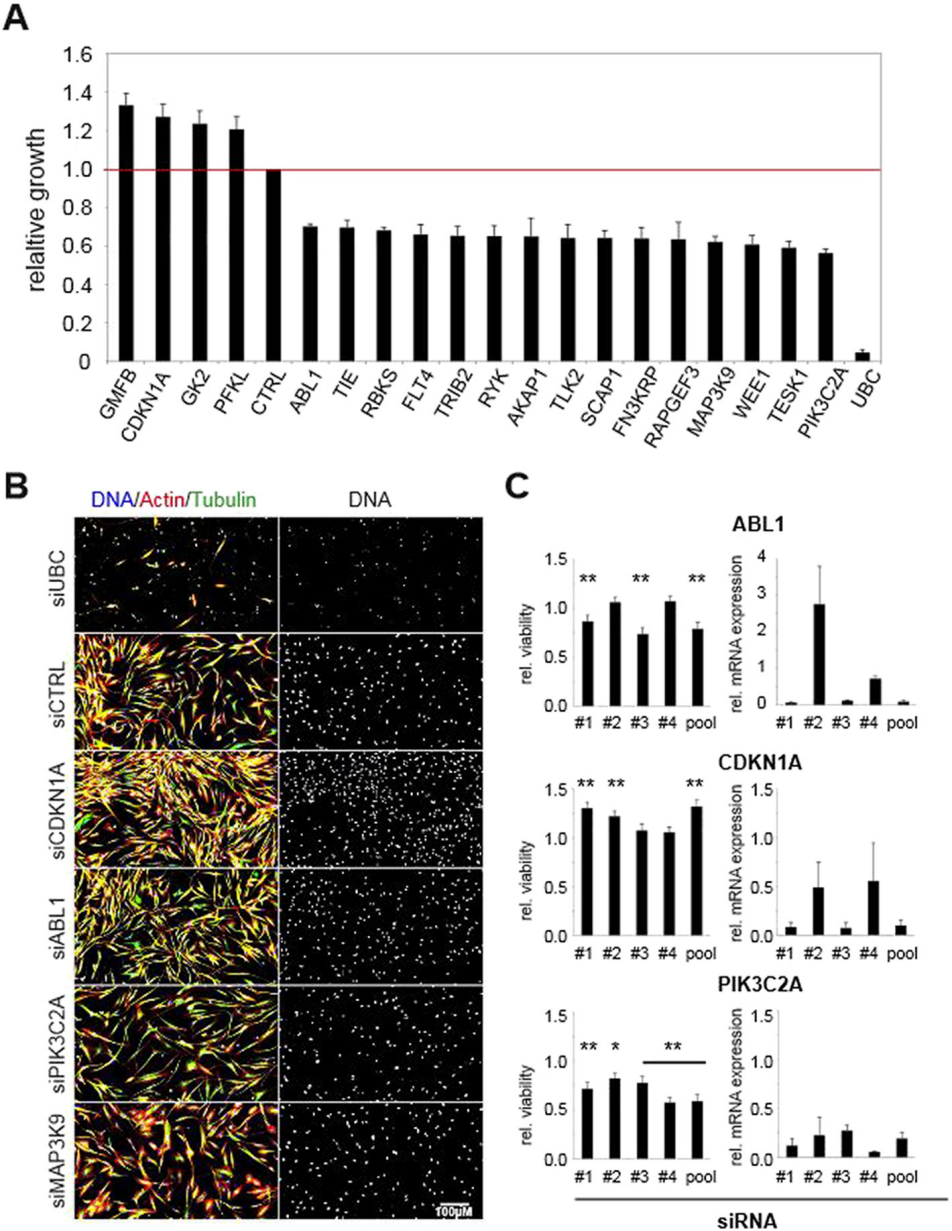
Validation of screening hits identified multiple kinases regulating MSC viability. **a** Cell viability was determined 72 h after siRNA reverse transfection (ATP level measured by luminescence) and the 19 genes which revealed the strongest phenotype are depicted. relative light units (RLU) were normalized to the negative control Rluc siRNA (red line). Data are presented as mean ± standard deviation (s.d.) of three screens. **b** Representative microscopy images of selected candidates revealed differences in morphology. Cells were reverse transfected with the corresponding siRNA and stained for microtubules (FITC), actin filaments (Alexa Fluor 547) and DNA (Hoechst). **c** Single siRNAs from selected candidates show good knockdown phenotype correlation. Single siRNAs and corresponding siRNA pools were analyzed for relative cell viability (CellTiterGlo) and knockdown efficiency (quantitative PCR) 72 h after reverse transfection. Relative viability compared to controls ± s.d. Significance of ATP level changes were calculated using unpaired two-tailed student’s *T*-test; n ≥ 3; *p ≤ 0.05; **p ≤ 0.01

We next chose 19 candidates that were associated with either an average increase of at least 20 % (a total of 4 genes) or a 25 % decrease in cell growth and viability (a total of 15 genes) (Additional file 1). We performed multiple independent retests (n ≥ 3) using the same assays in MSCs from different donors (Fig. 3), as well as laser scanning cytometry measuring DNA content (Additional file 2). These assays confirmed 12 out of 19 candidates from the initial screening experiment. The candidates included the known cell-cycle regulators ABL1, CDKNA1/p21 and WEE1, together with genes that were previously linked to viability, such as *PIK3C2A*. ABL1 and WEE1 regulate the G2/M checkpoint through interactions with CDC2 and retinoblastoma protein [23, 24], whereas CDKN1A/p21 regulates entry into G1 phase [25]. PIK3C2A belongs to the phosphoinositide 3-kinase (PI3K) family and plays a role in insulin signaling as well as dynamin-independent internalization pathways [26, 27]. *PIK3C2A* silencing reduces hepatoma cell proliferation and induces apoptotic cell death in a number of cancer cell lines [28, 29]. Overall, the homogenous cell growth and viability assay as well as the quantification by laser scanning cytometry yielded equivalent results which underlined the robustness of the screening platform in MSCs.

To detect additional phenotypes we used high content imaging by staining MSCs for actin, tubulin and DNA (Fig. 3b; Additional file 3). While mild viability phenotypes such as knockdown of ABL1 showed no obvious visual effect, stronger phenotypes such as knockdown of CDKN1A/p21 and PIK3C2A showed visual changes in cell and nuclear number. Interestingly, several siRNAs targeting *MAP3K9* and *TRIB2* showed distinct morphological phenotypes when compared with control MSCs. MAP3K9 is frequently mutated in metastatic melanomas, but its function remains unclear [30]. TRIB2 contains a Trb domain that lacks the active site lysine of protein serine-threonine kinases. It has multiple functions, including in lung tumorigenesis through down-regulation of C/EBPα, stimulation of interleukin-8 production by human monocytes and regulation of Toll-like receptor 5 signaling [31–33]. In summary, we could demonstrate that high-throughput screening and high content imaging can be combined to investigate morphological phenotypes in primary adult stem cells.

Next, we analyzed knockdown efficiency of individual siRNAs. We found that *ABL1* and *PIK3C2A* displayed a good correlation between mRNA knockdown and growth phenotypes (Fig. 3c). However, *CDKN1A*/p21, the cyclin-dependent kinase inhibitor 1A, did not show a strong correlation, which might indicate experimental variability or technical artifacts, differences in isoform targeting or off-target effects. In summary, we have confirmed our screening results, demonstrated the validity of our assay setup and have identified a number of kinases that were essential for maintaining MSC viability.

### *PIK3C2A* and *WEE1* silencing altered the cell cycle profile of MSCs

**Fig. 4:**
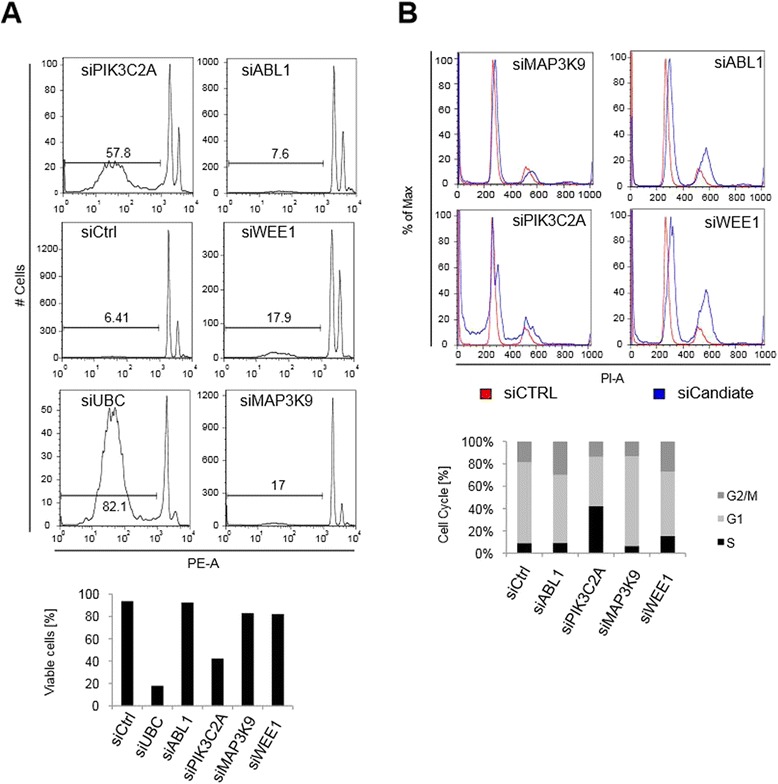
*PIK3C2A* and *WEE1* silencing alters the cell cycle profile of primary MSCs. Cytometric analysis of MSCs 96 h after siRNA treatment confirms viability phenotypes of selected candidates. **a** Representative fluorescence flow cytometry analysis of different MSC donors. MSCs were stained with 200 μg/ml of propidium iodide (PI), 0.1 %, phycoerythrin (PE), 0.1 %, sodium azide, 0.1 % Triton-X100 and 10 μg/ml RNAses for 2–4 h at 4 °C. Single cells were analyzed for fragmented DNA and dead cells were gated to quantify viable cells (PE-A). The percentage of viable cells was calculated from three biological replicates and average viability was plotted. **b** Representative histograms of MSCs analyzed with FlowJo 887 for sub G1, S and G2 peaks (PI-A). *PIK3C2A* and *WEE1* show a shift in DNA content (blue) compared with the negative control (red). Quantification of cell cycle percentages shows that *PIK3C2A* and *WEE1* silencing led to a significant (*p* ≤ 0.01) increase in S-phase and G2 peaks, respectively. Average of three biological replicates is shown and significant cell cycle changes were calculated using unpaired two tailed student’s *T*-test

Nuclear intensity and ATP content are two parameters that have been frequently used to determine cell viability. To assess whether the decrease in cell viability and nuclear intensity observed after RNAi treatment (Fig. 3) was caused by lower cell proliferation, decreased metabolism or apoptosis, the total percentage of dead cells was measured by fluorescence flow cytometry 96 h post-transfection. All tested candidates showed a significant decrease in cell number 96 h post-RNAi treatment (Fig. 4). Both PIK3CA2 and MAP3K9 were associated with weaker reduction in cell viability compared with the CTG-based re-tests (Fig. 3). Conversely, the phenotypes for ABL1 and WEE1 were more pronounced in the fluorescence flow cytometry analysis (Fig. 4). Cell cycle profiling revealed an increase in G2/M DNA content in *ABL1* (26 %) and *WEE1* (38 %) RNAi treated samples compared with 12 % in the control (Fig. 4). Although we observed an increase in the G2/M DNA content after both ABL1 and WEE1 ablation, only the increase in WEE1 proved statistically significant (*p* ≤ 0.05). In the case of PIK3CA2, 9 % of cells were scored to be in S-phase, which is significantly more compared with the 2 % measured in the control. These experiments further supported that the screening experiments have identified valid candidates. Several candidates showed severe cell cycle alterations in primary MSCs that led to the observed growth and viability effects.

### ‘Functional fingerprinting’ to identify MSC cell populations

The experiments described above identified individual candidate genes being involved in MSC growth and proliferation behavior. These genes were selected by means of their phenotypic strength and validated by independent assays. We could confirm their function in MSCs, but their functions are, in general, not specific to MSCs [24, 25]. We then applied an alternative strategy to identify a subset of genes that might functionally 'fingerprint' MSCs by comparing loss-of-function 'profiles' of MSCs with other primary cells and cell lines. This approach could then identify 'patterns' of multiple weaker phenotypes that together can be diagnostic for a particular cell state. This is similar to approaches that have been successfully used to classify cancer subtypes based on gene expression profiles. Here we have applied functional readouts with the goal to identify a network of functionally related genes that are characteristic for MSCs.

**Fig. 5:**
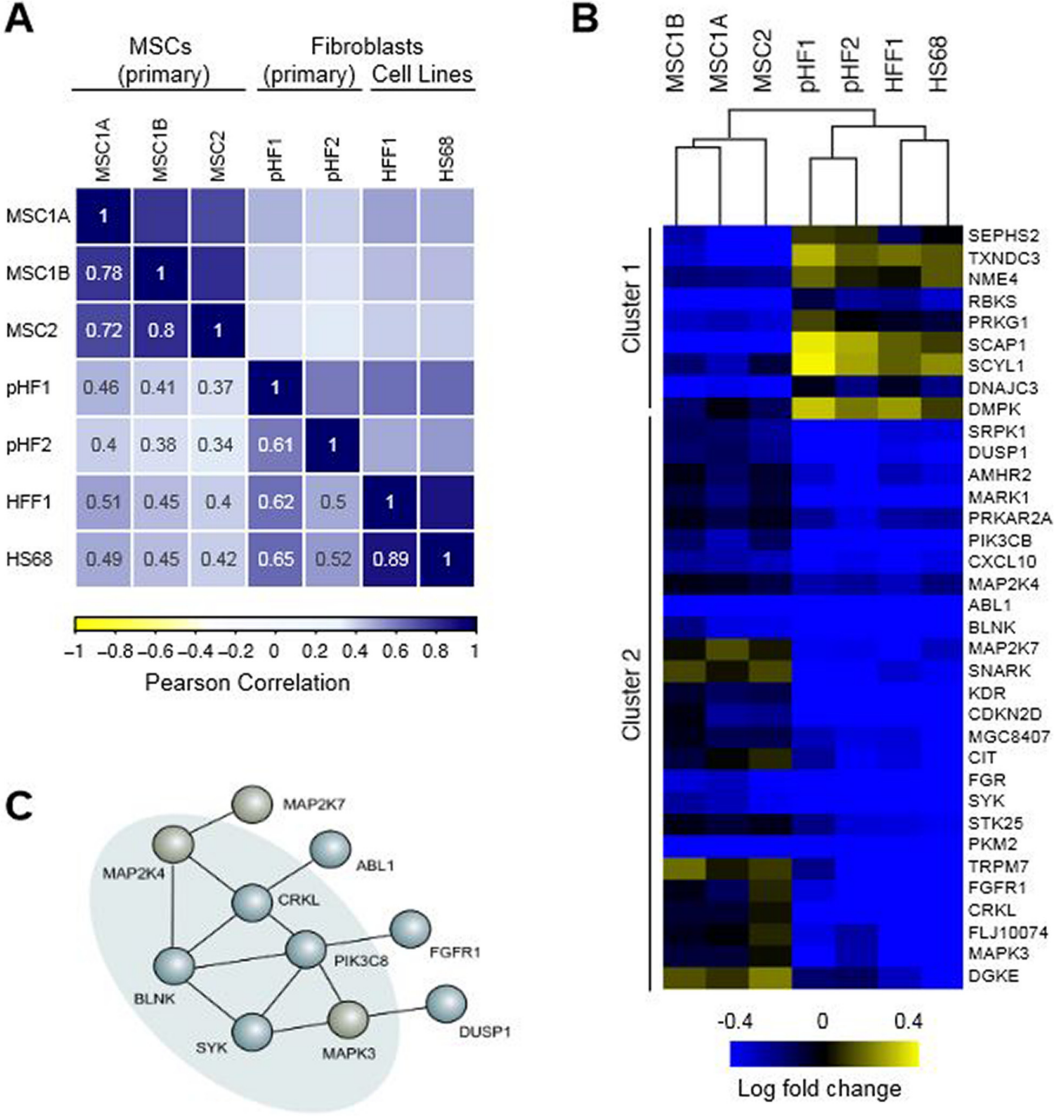
Kinome profiling identifies MSCs as a distinct cell population. **a** MSCs can be clustered by their unique kinome profile, which distinguishes them from primary fibroblasts and established fibroblast cell lines. Visual representation based on the Pearson correlation coefficient of log_2_ fold changes in growth and cell proliferation. Kinome data from primary fibroblasts (pHF1, pHF2) and fibroblast cell lines (HFF, HS68) and primary MSCs. **b** Bi-dimensional clustering of kinome-viability phenotypes from all screened cells reveals a set of 35 kinases with a differential effect on cell viability between MSCs and fibroblasts. **c** All kinases with a differential phenotype were analyzed by STRING (v.9.0) to create a curated interaction network [34]. Out of the 35 identified kinases, 12 form an interaction network which is enriched in B-cell-receptor signaling components

As the identity of MSC populations has been a controversial issue, we have compared kinome-wide perturbation data sets from MSC and fibroblasts. Under identical conditions, we screened two primary fibroblast isolations as well as two fibroblast cell lines (HFF1 and HS68). The data analysis was performed as described above. We then calculated the pair-wise Pearson correlation coefficients between the kinome-wide data sets. We found that MSC data sets showed high Pearson correlation coefficients among each other but not with any of the fibroblast data sets (Fig. 5a). Conversely, the fibroblast cell lines also correlated with each other, especially HFF1 and HS68 cells.

We then addressed the issue of whether we could identify a subset of genes that could distinguish MSCs obtained from different donors versus fibroblasts and fibroblast cell lines. This explorative approach identified 35 genes whose knockdown displayed differential effects on growth and viability of MSCs compared with fibroblasts (Fig. 5b; Additional file 4). Bi-dimensional clustering clearly separated MSCs and fibroblast lineages, with two predominant clusters that differed between both lineages. Subsequently, we tested whether our sample size allowed for conclusiveness and performed post hoc power calculations on the identified clusters. The calculations were based on the effect size and we assumed a significance level of 0.05. The sample size (N = 2 for MSCs and N = 4 for fibroblasts) in cluster 1 was sufficient to achieve a power above 90 % (1 - β ≥ 0.94) for all genes. We therefore conclude that our approach is able to identify clusters of genes that functionally distinguish MSCs from fibroblasts.

To further investigate the different sub-clusters, we functionally classified them based on our prior knowledge using STRING and other annotation information. This analysis showed that cluster 2 contained several genes enriched for B-cell-receptor (BCR) signaling components, centered around the Src family of protein tyrosine kinases ABL1, SYK and FGR (Fig. 5c). All these genes led to a slight, but measurable, impact on cell growth and viability of MSCs, whereas the same genes were associated with significantly stronger inhibition of the growth of fibroblasts. In contrast, cluster 1 contained genes that had a significantly stronger impact on MSCs than on fibroblasts. Taken together, the sub-clustering identified a network of genes that could distinguish between primary MSCs and fibroblasts.

## Discussion

We have demonstrated that a high-throughput RNAi screen approach was able to characterize donor-derived MSCs for genetic dependencies, and that despite cellular heterogeneity, quantitative functional genomic experiments were possible. Our high-throughput screening experiments have defined several classes of kinases that are essential for MSC expansion. MSC populations from the same tissue of origin have been described to be highly heterogeneous in their proliferative and differentiation behavior [35], posing a major challenge for cellular phenotyping approaches [17]. The lack of specific markers and their more general definition (according to a panel of certain characteristics by Dominici *et al*. in 2006 [11]) additionally hamper the transition from bench to bedside.

In this study, we have used MSCs derived from several healthy donors and have analyzed the screening outcomes of intra- and inter-biological replicates. We could show that MSC heterogeneity did not limit our ability to perform homogenous (such as cell viability) or single-cell analysis and to detect changes in morphology (by microscopy). We have demonstrated that assay performance was high — such as measured by replicate reproducibility and control performance — on par with similar assays performed in transformed cell lines. This high-throughput platform in primary MSCs has enabled us to quantitatively measure many phenotypes and the current data sets lay the methodological foundation for a broad range of homogenous or single-cell functional screens.

In our screen of kinases in the human genome, we have identified a phenotypic change for about 10 % of targeted kinases. For example, we identified cell cycle regulators such as WEE1 and ABL1, which regulate the G2/M checkpoint through interactions with CDC2 and retinoblastoma protein [24], and CDKN1A/p21, which regulates entry into G1 phase [25]. Furthermore, multiple genes have previously been associated with cell viability, such as *PIK3C2A* and *AKAP1*. PIK3C2A reduces hepatoma cell proliferation upon silencing [29]. AKAP1 binds to type I and type II regulatory subunits of protein kinase A and is potentially involved in cAMP signaling, thereby regulating malignant lymphocyte survival [36]. PIK3C2A has not been reported to be required for MSC proliferation previously.

To exclude potential off-target effects, four independent siRNA designs targeted against the same kinase of selected candidates were tested. ABL1 and PIK3C2A showed a complete and CDKN1A/p21 a partial phenotype knock-down correlation. Conversely, *AKAP1* silencing failed to reproduce the phenotype observed in the screen with single siRNA designs. The false discovery rate in MSCs was not higher than in previously performed screens in different cell lines [14, 16].

To validate our candidates by two orthogonal assays, we measured total DNA content and analyzed cell cycle profiles of MSCs by fluorescence flow cytometry analysis. DNA nuclear intensity resembled the effect strength of the ATP content-based luminescence CTG assay, thus confirming our candidates. In the case of ABL1 we observed G2/M cell cycle arrest and measured no significant levels of fragmented DNA. ABL1 localization and DNA binding were regulated during the cell cycle by the retinoblastoma protein and it was recently discovered that ABL1 might be a key component of the spindle orientation machinery [24, 37]. Previous studies already showed that its depletion in adherent cells caused abnormal rotation of the spindle [37]. G2/M cell cycle arrest, as described in this study, is therefore in line with the importance of ABL1 for chromosome segregation in MSCs.

The ability to distinguish MSCs from other primary cells has remained a significant challenge. Expression of surface markers varies *in vivo* and *in vitro* [38]. Furthermore, fibroblasts share most characteristics of MSCs as currently defined by the International Society of Cellular Therapy. With the exception of the functional assays, the current definition is not suitable to clearly discriminate between these two cell populations [11, 39, 40]. With our explorative approach we have developed a method that could be used to characterize and define MSCs. Bi-dimensional clustering of the scored viability phenotypes from the kinome-wide screens of MSCs and fibroblasts revealed that MSCs cluster significantly differently from all other tested cells. This supports the notion that MSCs are actually different from fibroblasts [12, 41, 42]. Once clinically significant fingerprints have been identified, respective protocols could be included as an additional quality control performed on a small aliquot of MSCs prior to their clinical application. Screening of MSC populations with a defined panel might further increase our understanding of their characteristics and clinical potential and could improve current standards of MSCs already used in clinical applications.

MSC and fibroblast physiologies seem to be influenced by different sets of kinases, thus confirming the diverse biological functions of fibroblast and MSCs. Our results indicate that a subset of kinases shows a MSC-specific viability profile after silencing. Furthermore, 13 kinases that form an interaction network enriched for BCR signaling components (SYK, FGR, BLNK, RKL, ABL1) and its downstream transducers (MAPK3, MAP2K7) were found [43–46]. Silencing all these kinases, except MAP2K7, induced a weaker viability phenotype in the MSC preparations compared with fibroblasts, suggesting that disruption of the BCR signaling pathway could either be compensated for by the MSCs or that the pathway was not essential for their survival. MSCs possess immunomodulatory functions and repress both T- and B-cell proliferation and maturation *in vitro* [47]. Stimulation of MSCs with the prototypic Toll-like receptor 2 ligand Pam3Cys has been reported to induce proliferation of MSCs [48], an effect of the BCR signaling pathway on MSC differentiation and proliferation has not yet been reported. Nevertheless, MSCs express a unique BCR signaling component which leads to G1 growth arrest when transfected into HEK 293T cells [49]. It can be speculated that the BCR pathway could play an additional role in MSC regulation and their immunomodulatory activities.

## Conclusions

In summary, we show that screening in heterogeneous primary adult stem cell populations is feasible and can be used to generate functional fingerprints. Furthermore, functional profiling of kinases can be used to define MSC populations and discriminate them from other cell types such as fibroblasts. Such functional profiling might be used reliably to supplement current characterization criteria. This explorative approach has therefore demonstrated that this method is in principle feasible to identify diagnostic fingerprints for different adult stem cell populations. Whether the observed profile is unique to MSCs isolated from bone marrow or whether this profile can be found in other MSC preparations derived from adipose tissues and other MSC sources remains to be established. Nevertheless, the present screening protocol could be used in the future to define different MSC populations based on a defined gene panel. This could provide significant criteria to supplement the current definition of MSCs. In conclusion, we have established a robust high-throughput screening platform that could be extended, e.g., to image-based screens or to map genotype-to-phenotype relationships [50] in primary preparations of MSCs.

## Additional files

Additional file 1: Table S1. List of the 19 candidate genes that were associated with, either an increase or decrease in cell growth and viability. Relative viability (ATP-based CellTiterGlo assay) after pooled siRNA knock-downs of the 19 candidate genes over the 3 screens and their standard deviation. Significance was calculated using student’s T-test, *n = 3*. Additional file 2: Figure S1. High-content microscopy confirms observed phenotypes and identifies morphological deviations in MSCs at the single cell level. **A** Measurement of DNA content through quantification of Hoechst staining intensities (white) confirms viability phenotypes observed in the ATP-based CellTiterGlo assay (black). The red line indicates normalized viability of the untreated MSCs. Significance was calculated using student’s *T*-test, N ≥ 3. **B** Morphological differences between the silenced kinases and untreated MSCs. Cells were reverse transfected with the according siRNA and stained for microtubules (FITC), actin filaments (Alexa Fluor 547 Phalloidin) and DNA (Hoechst). Additional file 3: Figure S2. Validation of remaining candidates identified additional kinases regulating MSC viability. **A** Average relative viability (CellTiterGlo assay) over three biological replicates of deconvoluted siRNA pools from the remaining candidates in relation to the relative knockdown efficiencies evaluated by quantitative PCR. **B** Remaining candidates for which the knockdown could not be confirmed. Relative viability compared with controls ± standard deviation. Significance of ATP level changes were calculated using unpaired two tailed student’s *T*-test: N *≥ 3*, **p* ≤ 0.05, ***p* ≤ 0.01. Additional file 4: Figure S3. Unsupervised clustering reveals significant differences between the kinome-viability profile of MSCs and fibroblasts. **A** MSCs can be grouped by their unique kinome profile, which distinguishes them from primary fibroblasts and established fibroblast cell lines. Bi-dimensional clustering of the complete kinome viability profiles from all screened cells. Phenotypes were clustered according to log2 fold changes of cell viability. Cell viability was measured by total ATP levels using the CellTiterGlo assay. Kinome viability data from primary fibroblasts (pHF1, pHF2) and fibroblast cell lines (HFF, HS68) versus primary MSCs.

## Abbreviations

BCR: B-cell receptor
CTG: CellTiterGlo
DMEM: Dulbecco's modified Eagle's medium
FCS: fetal calf serum
FITC: fluorescein isothiocyanate
MSC: mesenchymal stem/stromal cell
PBS: phosphate buffered saline
Pen/Strep: penicillin/streptomycin
RNAi: RNA interference
siRNA: small interfering RNA
