# Supplementary material for: Functional fingerprinting of human mesenchymal stem cells using high-throughput RNAi screening
Source: Genome Med. 2015 May 17;7(1):46. doi: 10.1186/s13073-015-0170-2 (PMC4481116; doi:10.1186/s13073-015-0170-2)
Supplement: Additional file 1: Table S1. — List of the 19 candidate genes that were associated with, either an increase or decrease in cell growth and viability. Relative viability (ATP-based CellTiterGlo assay) after pooled siRNA knock-downs of the 19 candidate genes over the 3 screens and their standard deviation. Significance was calculated using student’s T-test, n = 3. [file 13073_2015_170_MOESM1_ESM.pdf]

**Table S1: Candidate gene list.**

| GENE | MSC1A | MSC1B | MSC2 | Average | STDEV | T-TEST |
|---|---|---|---|---|---|---|
| **GMFB** | 1.38 | 1.26 | 1.36 | 1.33 | 0.06 | 0.01 |
| **CDKN1A** | 1.28 | 1.33 | 1.20 | 1.27 | 0.06 | 0.02 |
| **GK2** | 1.31 | 1.18 | 1.22 | 1.24 | 0.07 | 0.03 |
| **PFKL** | 1.26 | 1.13 | 1.23 | 1.21 | 0.07 | 0.03 |
| **CTRL** | 1.00 | 1.00 | 1.00 | 1.00 | | |
| **ABL1** | 0.71 | 0.71 | 0.69 | 0.70 | 0.01 | 0.00 |
| **TIE** | 0.66 | 0.72 | 0.71 | 0.70 | 0.04 | 0.00 |
| **RBKS** | 0.67 | 0.70 | 0.68 | 0.68 | 0.02 | 0.00 |
| **FLT4** | 0.67 | 0.71 | 0.61 | 0.66 | 0.05 | 0.01 |
| **TRIB2** | 0.60 | 0.70 | 0.66 | 0.65 | 0.05 | 0.01 |
| **RYK** | 0.60 | 0.65 | 0.71 | 0.65 | 0.05 | 0.01 |
| **AKAP1** | 0.73 | 0.68 | 0.54 | 0.65 | 0.10 | 0.02 |
| **TLK2** | 0.56 | 0.67 | 0.69 | 0.64 | 0.07 | 0.01 |
| **SCAP1** | 0.60 | 0.64 | 0.68 | 0.64 | 0.04 | 0.00 |
| **FN3KRP** | 0.58 | 0.69 | 0.65 | 0.64 | 0.06 | 0.01 |
| **RAPGEF3** | 0.53 | 0.68 | 0.69 | 0.63 | 0.09 | 0.02 |
| **MAP3K9** | 0.61 | 0.65 | 0.60 | 0.62 | 0.03 | 0.00 |
| **WEE1** | 0.66 | 0.60 | 0.56 | 0.61 | 0.05 | 0.00 |
| **TESK1** | 0.55 | 0.62 | 0.60 | 0.59 | 0.03 | 0.00 |
| **PIK3C2A** | 0.56 | 0.59 | 0.54 | 0.56 | 0.02 | 0.00 |
| UBC | 0.05 | 0.06 | 0.03 | 0.05 | 0.02 | 0.00 |
