# Supplementary figures and images for: Functional fingerprinting of human mesenchymal stem cells using high-throughput RNAi screening
Source: Genome Med. 2015 May 17;7(1):46. doi: 10.1186/s13073-015-0170-2 (PMC4481116; doi:10.1186/s13073-015-0170-2)

**A**

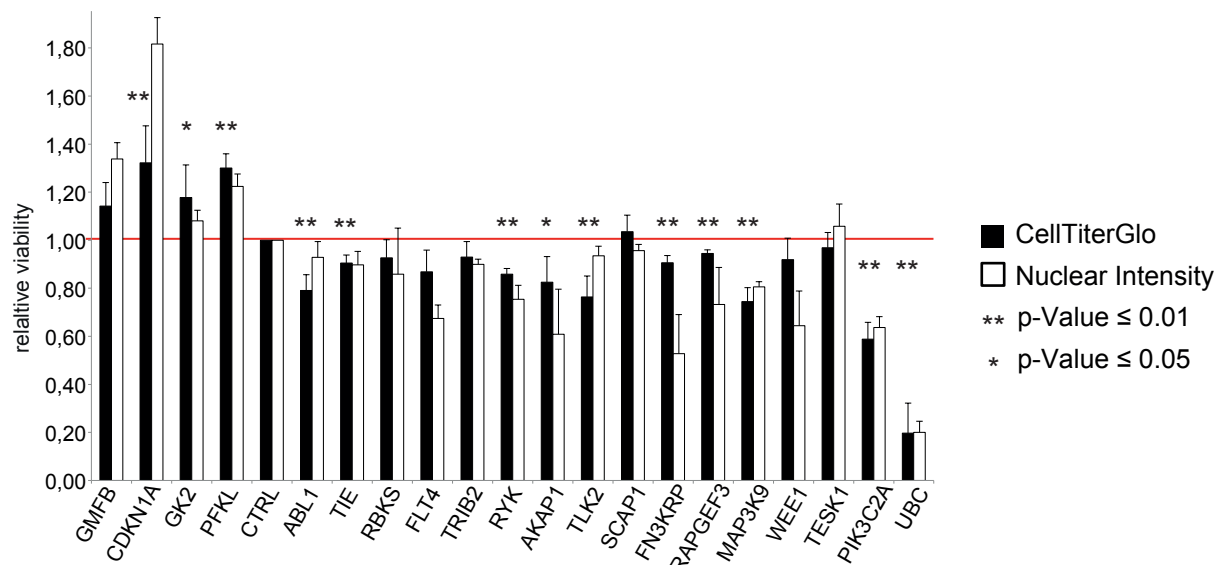

**B**

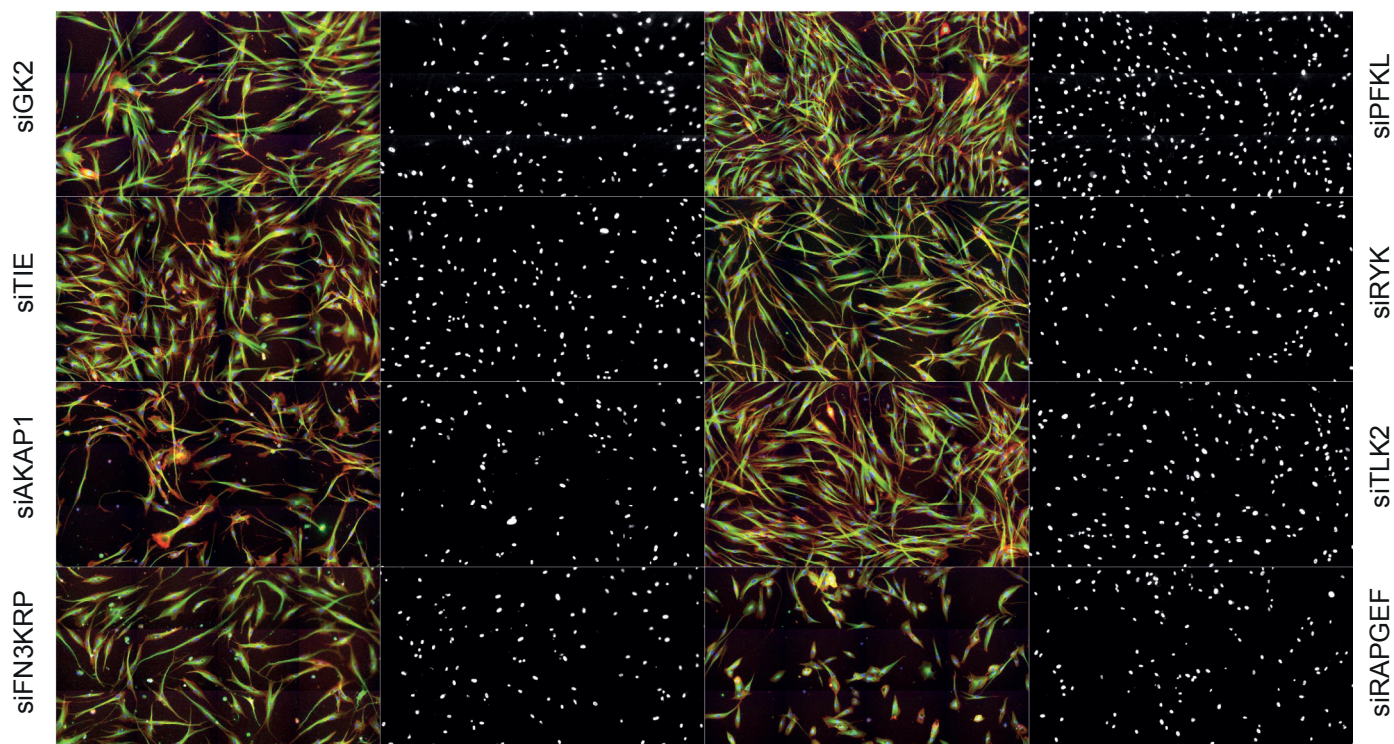

Supplement: Additional file 2: Figure S1. — High-content microscopy confirms observed phenotypes and identifies morphological deviations in MSCs at the single cell level. A Measurement of DNA content through quantification of Hoechst staining intensities (white) confirms viability phenotypes observed in the ATP-based CellTiterGlo assay (black). The red line indicates normalized viability of the untreated MSCs. Significance was calculated using student’s T-test, N ≥ 3. B Morphological differences between the silenced kinases and untreated MSCs. Cells were reverse transfected with the according siRNA and stained for microtubules (FITC), actin filaments (Alexa Fluor 547 Phalloidin) and DNA (Hoechst). [file 13073_2015_170_MOESM2_ESM.pdf]

A

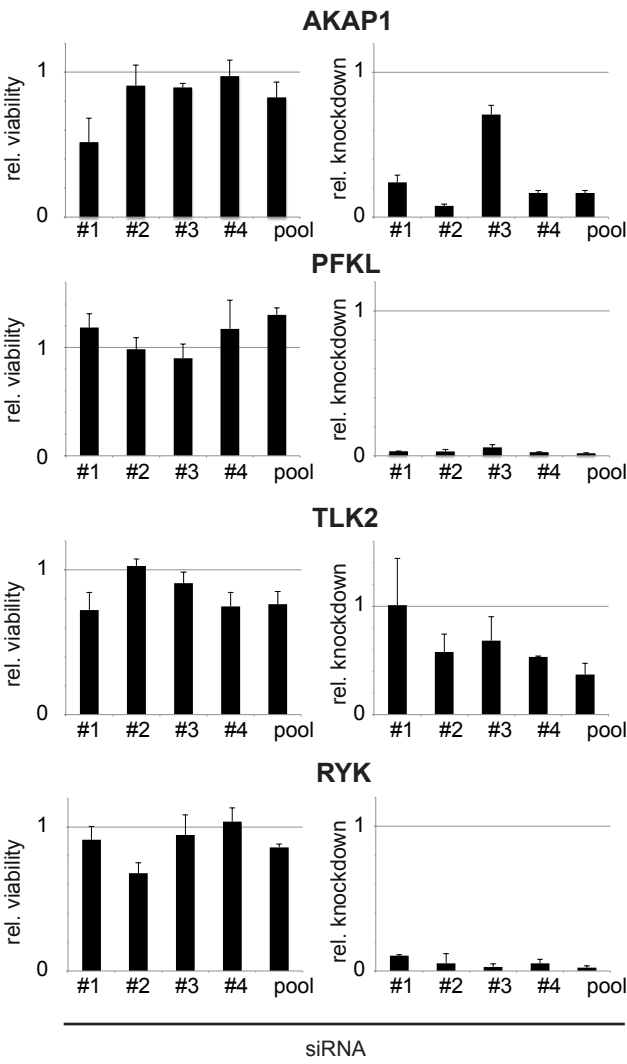

B

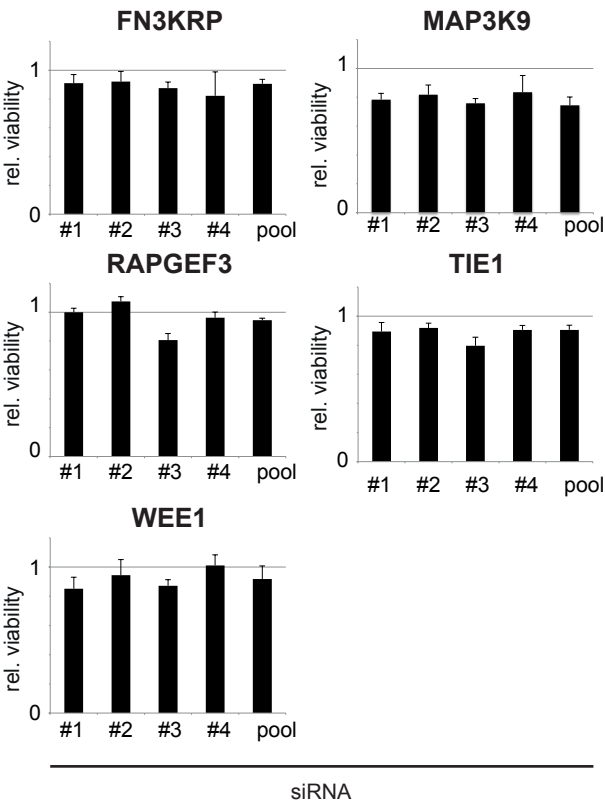

Supplement: Additional file 3: Figure S2. — Validation of remaining candidates identified additional kinases regulating MSC viability. A Average relative viability (CellTiterGlo assay) over three biological replicates of deconvoluted siRNA pools from the remaining candidates in relation to the relative knockdown efficiencies evaluated by quantitative PCR. B Remaining candidates for which the knockdown could not be confirmed. Relative viability compared with controls ± standard deviation. Significance of ATP level changes were calculated using unpaired two tailed student’s T-test: N ≥ 3, *p ≤ 0.05, **p ≤ 0.01. [file 13073_2015_170_MOESM3_ESM.pdf]

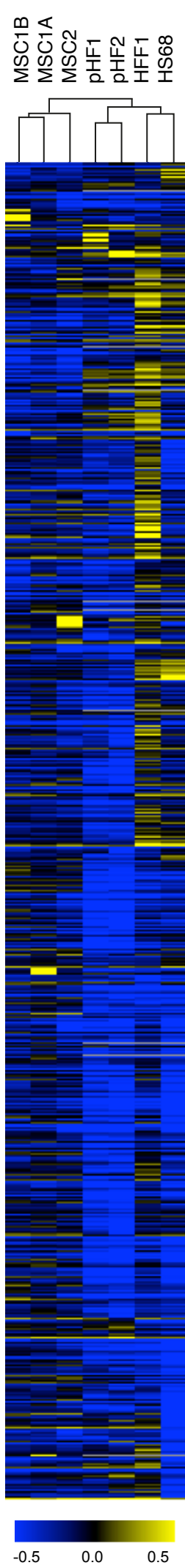

Supplement: Additional file 4: Figure S3. — Unsupervised clustering reveals significant differences between the kinome-viability profile of MSCs and fibroblasts. A MSCs can be grouped by their unique kinome profile, which distinguishes them from primary fibroblasts and established fibroblast cell lines. Bi-dimensional clustering of the complete kinome viability profiles from all screened cells. Phenotypes were clustered according to log2 fold changes of cell viability. Cell viability was measured by total ATP levels using the CellTiterGlo assay. Kinome viability data from primary fibroblasts (pHF1, pHF2) and fibroblast cell lines (HFF, HS68) versus primary MSCs. [file 13073_2015_170_MOESM4_ESM.pdf]
